# The challenging task to select *Salmonella* target serovars in poultry: the Italian point of view

**DOI:** 10.1017/S0950268821001230

**Published:** 2021-05-24

**Authors:** Marta Leati, Alessandra Zaccherini, Luigi Ruocco, Stefania D'Amato, Luca Busani, Laura Villa, Lisa Barco, Antonia Ricci, Veronica Cibin

**Affiliations:** 1 Istituto Zooprofilattico Sperimentale delle Venezie, Legnaro, PD, Italy; 2 Istituto Zooprofilattico Sperimentale del Mezzogiorno, Portici, NA, Italy; 3 Italian Ministry of Health, Rome, Italy; 4 Istituto Superiore di Sanità, Rome, Italy

**Keywords:** Epidemiology, Poultry, Public health, *Salmonella*

## Abstract

According to the European Food Safety Authority (EFSA) and European Centre for Disease Prevention and Control (ECDC) annual report, human salmonellosis is mostly related to consumption of contaminated poultry products. Since 2003 in Europe, the *Salmonella* serovars considered relevant for human health and subject to control in breeding hens of *Gallus gallus* are: *S*. Enteritidis, *S*. Typhimurium (including the monophasic variant), *S*. Infantis, *S*. Hadar and *S*. Virchow. Herein, we investigated the Italian epidemiological situation from 2016 to 2018, comparing *Salmonella* serovar distributions in humans and poultry, in order to identify the target *Salmonella* serovars that, if controlled, would potentially have the largest public health impact in Italy. The results showed that control of *S*. Virchow and *S*. Hadar does no longer seem to be a priority in Italy and that *S*. Napoli and *S*. Derby, which are not included in the group of EU target serovars, are among the most frequent serovars isolated from humans in Italy. While *S*. Derby has its main reservoir in pigs, *S.* Napoli does not have a specific reservoir. However, because this serovar is frequently isolated from breeding poultry flocks and is characterised by causing severe human illness, it is a potential target *Salmonella* serovar in breeding hens of *Gallus gallus* in Italy.

Salmonellosis is the second most common foodborne infection in Europe, with over 90 000 confirmed human cases per year, but in Italy, according to the reporting system in place, it is the most common foodborne infection, with more than 3500 human cases reported per year [1]. Food products derived from poultry, eggs mostly, are the types of food more frequently implicated in salmonellosis foodborne outbreaks [1], and thus, Regulation (CE) 2160/2003 was implemented with the aim of reducing human exposure to contaminated food. According to this Regulation, Member States (MSs) have to put in place national control programmes (NCPs) aiming to meet the target of 1% prevalence in *Gallus gallus* breeding flocks for target *Salmonella* serovars that are considered relevant for public health. Indeed, considering the pyramidal structure of poultry production, it is expected that the application of sanitary measures at the apex of the pyramid (breeders) would provoke effects also at the base (broilers and laying hens). More specifically, for all MSs, the following target *Salmonella* serovars were initially identified: *S*. Enteritidis, *S*. Typhimurium (including the monophasic variant), *S*. Infantis, *S*. Hadar and *S*. Virchow.

On 16 January 2019, EFSA published a Scientific Opinion on ‘*Salmonella* control in poultry flocks and its public health impact’ that evaluated the impact on human salmonellosis cases if the conventional EU targets for *Salmonella* in breeding hens were to be changed. The need to review the target serovars is based on the observation that from 2012 onwards, no significant decrease of human salmonellosis cases was observed [2]. In the 2019 Scientific Opinion, two main scenarios were taken into account: scenario 1, targeting all *Salmonella* serovars and scenario 2, identifying new specific target serovars, including variable ones, based on the occurrence and epidemiology of each MS. In particular, within scenario 2, the hypothesis of keeping *S.* Enteritidis, *S*. Typhimurium (including the monophasic variant) and *S*. Infantis as target serovars and replacing *S*. Virchow and *S*. Hadar with others was explored. This last option is justified mainly by the distribution of the most frequent serovars that are reported in human cases in the EU [1]. *S*. Kentucky was proposed as a fourth target serovar, and for a fifth target serovar, different options were discussed, among them, *S*. Thompson, *S.* Heidelberg and a fifth serovar optionally chosen by each MS. These serovars were proposed according to different criteria, including antimicrobial resistance characteristics, potentiality to be transmitted vertically in animals and virulence [2].

Herein, we explored the current epidemiological situation in Italy in order to assess these assumptions and identify the target serovars with the highest potential to effectively impact national public health.

The identification of target serovars was based on the evaluation of data collected in the framework of Directive 2003/99/EC from 2016 to 2018. In detail, NCPs data, extracted from the National Informative System [3], that provides flock prevalences and serovar distributions, and data on human isolates, collected by the ENTER-NET surveillance network and provided to the Ministry of Health, were used. ENTER-NET is a laboratory-based surveillance of enteric bacterial pathogens coordinated by the Istituto Superiore di Sanità that involves more than 140 clinical microbiology laboratories covering about 65% of the Italian territory.

During the three years under observation (2016–2018), the flock-level prevalences of *Salmonella* in breeding hens of *Gallus gallus* (both in laying and rearing phases) in Italy were 0.9% in 2016, 2.6% in 2017 and 1.8% in 2018. The six most recurrent serovars in this animal category in the considered period were: *S.* Veneziana, accounting for 21% of all the detected isolates, followed by *S.* Mbandaka (17.6%); *S.* Kedougou, (6.2%); *S.* Infantis (5.5%); *S.* Typhimurium (4.0%) and *S.* Napoli (3.9%). Regarding the current EU target serovars, the frequency of detection in the same time window, except for *S.* Typhimurium and *S.* Infantis, was as follows: 1.6% for the monophasic variant of *S.* Typhimurium and 1.3% for *S*. Enteritidis, while *S.* Virchow and *S.* Hadar were never detected. Regarding the optional new target serovars hypothesised by the EFSA, in Italy, the frequency of detection fluctuated over the considered period: for *S.* Kentucky in breeding hens of *Gallus gallus*, it was 8.1% in 2017, but this serovar was never detected in 2016 or 2018; for *S.* Thompson it was 6.1% in 2017, 5.5% in 2018 and no detections at all in 2016; *S*. Heidelberg constituted 6.6% of all serovars in 2016 and 2.0% in 2017, while in 2018, this serovar was never detected.

Exploring the serovar distribution at the base of the poultry production pyramid, the following situation was observed. *S.* Infantis was the most frequently detected serovar in broilers, accounting for more than 50% of all the isolates, and in laying hens was the third most common serovar, accounting for 5.5% of all isolates. *S.* Enteritidis was the second most common serovar (11.6%) in laying hens, but was very infrequently detected in broilers, accounting for barely 0.1% of all isolates detected during the considered period. *S*. Typhimurium and its monophasic variant in broilers, and Typhimurium monophasic variant in laying hens were very seldom detected over the three years of observation, while *S.* Typhimurium was occasionally detected in laying hens.

Regarding *S*. Virchow and *S.* Hadar, a similar situation was observed in laying hens (one flock positive for each serovar, corresponding to an average frequency of detection of 0.32% and 0.17%, respectively) and in broilers (one positive flock for *S.* Virchow, equal to 0.01% of all isolates identified).

Regarding *S.* Heidelberg, in laying hens, a situation similar to that in breeding hens (almost no positive flocks) was observed, while for *S.* Thompson and *S.* Kentucky, the situation was very different. *S.* Thompson constituted 3.6%, 3.2% and 1.6% of all the isolates in 2016, 2017 and 2018, respectively; *S.* Kentucky, accounting for about 40% of all the identified isolates each year, was the most frequently detected serovar by far. In broilers, we found that, while frequencies of detection of *S.* Kentucky and *S*. Heidelberg were always below 1%, *S*. Thompson was consistently the second most common serovar, corresponding to 15.3% of all isolates detected in 2016, 18.7% in 2017 and 8.8% in 2018.

Observing the distribution of serovars collected by the ENTER-NET network from humans in Italy in the same timeframe (2016–2018), the serovars detected with a higher frequency were: the monophasic variant of *S*. Typhimurium comprising 35.0% of all the identified isolates, *S.* Enteritidis comprising 14.7%, *S*. Typhimurium 10.2%, *S.* Napoli 4.9%, *S*. Derby 3.0% and *S.* Infantis 2.7% of all reported isolates. Each of the other serovars detected during 2016–2018 was reported with a frequency below 2.5%. *S.* Hadar and *S*. Virchow, the other current target serovars recommended for breeding flocks of *Gallus gallus,* together accounted for 0.36% of all detected human isolates. The epidemiological situation was slightly different when we considered the proposed alternative serovars. The frequency of detection of *S.* Heidelberg was very low (it accounted for 0.05% of all human isolates during the three years), while *S.* Kentucky and *S.* Thompson were also detected at low frequencies, 0.24% and 0.9%, respectively.

Considering the data described herein, the hypothesis to expand targets in breeding flocks of *Gallus gallus* to all *Salmonella* serovars must be considered excessive, at least in Italy.

*S.* Typhimurium and *S.* Derby, which in Italy are among the most frequently isolated serovars from humans, have their principal reservoirs in swine [4, 5]. Moreover, it has already been demonstrated that in Italy, human cases are mostly associated with the pig chain [6–8]. Therefore, the use of significant resources for further decreasing the *Salmonella* prevalence in breeding flocks of *Gallus gallus* is unlikely to result in a substantial decrease in the number of human cases in our country.

Considering the frequency of detection in Italy of *S*. Virchow and *S*. Hadar in both poultry and in humans, the hypothesis to not include these as target serovars would not plausibly affect the number of human cases. Regarding the proposal to include *S.* Kentucky as the fourth target serovar, even though we did not observe a high frequency of detection in breeding hens, we did verify the importance of this serovar in laying hens. Therefore, we could expect that targeting this serovar at the European level could have a beneficial effect in terms of public health also in Italy, even though this is difficult to quantify at the moment.

As far as the choice of the fifth target serovar is concerned, between *S*. Thomson and *S*. Heidelberg, based on the observed data, we recommend *S*. Thompson. However, if we could autonomously identify the fifth target serovar according to our epidemiological situation, we would suggest *S*. Napoli should be included. This serovar has a low prevalence in most EU countries, both in breeding hens, where it does not appear among the 20 most frequently detected serovars, and in humans, where it comprises only about 0.4% of confirmed cases [2]. However, this is not the case in Italy, where the frequency of detection of this serovar is noteworthy in breeding hens and is nearly 10 times higher in humans than in the rest of Europe.

According to the aforementioned considerations, we expect that control of *S.* Napoli in breeding hens of *Gallus gallus* in Italy could likely lead to a significant reduction in the number of human salmonellosis cases. Regarding *S.* Napoli, it is important to point out that in Italy, this serovar has been growing in importance since 2000 [5, 9], and that despite the efforts to clarify its epidemiology, the source of infection for humans remains unclear. So far, it has been strongly associated with environmental sources [5]. However, the data presented here confirm that this serovar does indeed occur in breeding birds at the top of the poultry production pyramid. Another important factor deserving particular attention is that *S*. Napoli has been associated with typhoidal serovars thanks to their genetic similarities, and hence, its control along the food chain is of paramount importance [10].

To conclude, the possibility of choosing one specific target serovar at country level, additionally to those considered relevant for all EU countries, could be an effective strategy. EU countries could be stimulated to provide data on their specific epidemiological situation, so giving EU risk managers the possibility to evaluate the effectiveness of this strategy in terms of cost−benefit ratio and in terms of implication for EU trade. Indeed, the in-depth evaluation of the epidemiological situation at country level could provide, as in this case, suggestions for appropriate allocation of the national public health resources, in order to achieve a lower number of human salmonellosis cases, with an undoubted benefit at community level.

## Data Availability

Data supporting the findings presented in this manuscript are described in the Italian reports on trend and sources of zoonoses and zoonotic agents in foodstuff, animals and feeding stuffs, that are accessible via EFSA website https://www.efsa.europa.eu/en/biological-hazards-data/reports Data on Salmonella serovars are accessible via ECDC website: https://atlas.ecdc.europa.eu/public/index.aspx?Dataset=27&HealthTopic=46%22.
